# Circular RNA circ_0067741 regulates the Hippo/YAP pathway to suppress lung adenocarcinoma progression by targeting microRNA-183-5p

**DOI:** 10.1080/21655979.2022.2060901

**Published:** 2022-04-17

**Authors:** Jianming Mo, Hao Nie, Chao Zeng, Hui Han, Ping Xu, Xingyuan Shi

**Affiliations:** a Department of Pulmonary and Critical Care Medicine, Peking University Shenzhen Hospital, Shenzhen, Guangdong, China; b Department of Radiation Oncology, The Fifth Hospital of Guangzhou Medical University, Guangzhou, Guangdong, China

**Keywords:** Circ_0067741, lung adenocarcinoma, Hippo/YAP pathway, miR-183-5p, LATS1

## Abstract

To discuss the effect and molecular mechanism of circular RNA circ_0067741 on the occurrence and development of lung adenocarcinoma (LUAD). QRT-PCR was utilized to detect circ_0067741, microRNA-183-5p (miR-183-5p) and large tumor suppressor 1 (LATS1) expressions in tumor tissues of 30 LUAD patients and LUAD cell lines (A549, Calu-3, H1299 and H1975). After overexpression or knockdown of circ_0067741-1 or miR-183-5p in H1299 and A549 cells, respectively, cell proliferation, viability, apoptosis, invasion and migration ability and angiogenesis ability were detected by MTT, cell cloning, flow cytometry, transwell and tube formation assays, respectively. The targeted relationship between miR-183-5p and circ_0067741 or LATS1 was validated using dual-luciferase reporter assay. We found that circ_0067741 expression was notably declined in LUAD cells and tissues. Overexpression of circ_0067741 inhibited the proliferation, migration, invasion, and angiogenesis of LUAD cells and promoted apoptosis. Moreover, circ_0067741 could sponge miR-183-5p to regulate LATS1 expression and then activate the Hippo/YAP pathway. Downregulation of LATS1 reversed the effects of circ_0067741 on the Hippo/YAP pathway and LUAD cells progression. In conclusion, circ_0067741 sponges miR-183-5p, and regulates LATS1 to activate Hippo/YAP pathway, thereby inhibiting the process of LUAD cells. And the circ_0067741/miR-183-5p/LATS1 axis can be a potential target for early diagnosis and targeted treatment of LUAD.

## Graphical Absract

**Figure:**
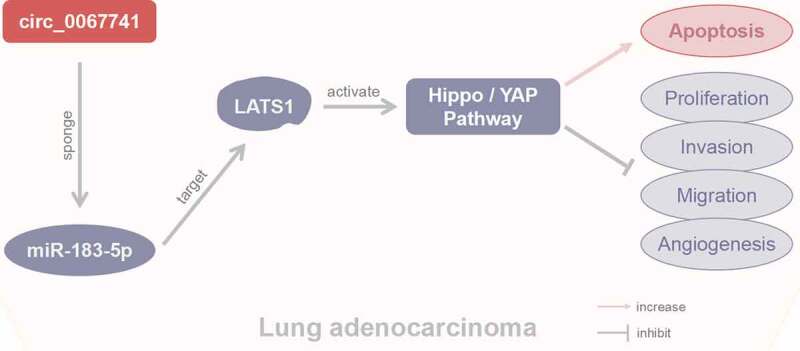

## Highlights

1. Circ_0067741 expressions are down-regulated in lung adenocarcinoma (LUAD) cells and tissues.
2. Overexpression of circ_0067741 inhibits LUAD cells progression.
3. Circ_0067741 inhibits LUAD progression via the miR-183-5p/LATS1 axis and activates the Hippo/YAP pathway.

## 1. Introduction

As one of the most prevalent malignancies, lung cancer is a deadly disease with a 5-year survival rate of approximately 21% [1]. And Lung adenocarcinoma (LUAD), accounting for approximately 40% of lung malignancies, is the most known subtype of lung cancer [2]. In recent decades, with the increasing incidence year by year, LUAD has become the highest incidence type in non-small cell lung carcinoma [3,4]. For now, major effective therapies for lung cancer include targeted treatment, immunotherapy and surgical resection. Nevertheless, because of the shortage of appropriate means of early diagnosis, local invasion or distant metastasis already occurred in most patients at the time of diagnosis. Usually, such patients have a poor prognosis and recovery [5]. Therefore, it is urgent to research sensitive diagnostic markers and the molecular mechanisms of the development of LUAD.

Circular RNA (circRNA), a closed circRNA, belongs to non-coding RNA, which can be stably presented in mammals. CircRNAs, which have neither a 5 ‘terminal cap nor 3’ terminal tail, can be composed of exon or intron splicing [6] with the features of conservation, high stability and specific expression [7,8]. Selectively conserved microRNA (miRNA) target sites exist in circRNAs, allowing circRNAs to be performed as the sponges of miRNA to regulate gene expression [9]. Recently, a great many researches have proved that circRNA adsorb miRNA to regulate target gene expression at the post-transcriptional level [10,11], thereby participating in human disease progressions including cancer. For instance, Zhang et al. demonstrated that circ_0007618 and circ_0029426 may be new biomarkers for the diagnosis and prognosis of LUAD [12]. Circ_0007142/miR-186/FOXK1 axis can promote LUAD progression [13]. Silencing circ_0020850 can inhibit LUAD progression by regulating the miR-195-5p/IRS2 axis [14]. Thus, circRNA is also one of the potential targets for the diagnosis and treatment of cancers. Circ_0067741 (Site: chr3:152,017,193–152,183,569) is a newly discovered circRNA. At present, the research on circ_0067741 is few, and the role and mechanism of circ_0067741 in LUAD have not been studied. MiR-183-5p, which locates in chromosome 7q32, is part of the miR-183 family, with a high degree of homogeneity [15]. Some researchers have indicated that the abnormal expression of miR-183-5p in LUAD is correlated with the development and poor prognosis of LUAD [16]. However, the association between circ_0067741 and miR-183-5p in LUAD remains unknown.

The Hippo/YAP signaling pathway, with a kinase cascade (composed by LATS1/2 and MST1/2) as the core, can regulate cell growth and proliferation [17]. In time of the activation of Hippo pathway, phosphorylation and activation of MST1/2 by MST1/2 can phosphorylate YAP and TAZ proteins. And after phosphorylation, TAZ/YAP translocate to the cytosol and undergoes ubiquitin-dependent degradation [18]. Defects of Hippo signaling pathway and excessive activation of YAP/TAZ are usual in all sorts of cancers, for example, the Hippo pathway promotes metastasis and castration resistance in prostate cancer [19]. In addition, it has also been reported that the upregulation of CD109 can promote epithelial-to-mesenchymal transition and stemness characteristics in LUAD by activating Hippo/YAP signaling [20].

Therefore, the aim of this study was to clarify circ_0067741 expression and investigate the role and mechanism of circ_0067741 and miR-183-5p in LUAD, thereby providing new potential targets for the therapy of LUAD.

## 2. Materials and methods

### 2.1 Clinical samples

Tumor tissues and corresponding adjacent non-cancerous tissues were obtained from 30 patients with LUAD who did receive neither chemotherapy nor radiotherapy before surgery in our hospital. Three independent pathologists were set for the identification of LUAD tissues. The Ethics Committee of the Peking University Shenzhen Hospital approved this study, and this study was conducted based on the guidance of the Declaration of Helsinki. Informed consents were signed by all subjects.

### 2.2 Cell culture

LUAD cell lines (Calu-3, A549, H1975, H1299) and human normal bronchial epithelial cell (HBE) were purchased from Shanghai Cell Research Institute, China. And 1640 or MEM medium (Gibco, USA) holding 10% fetal bovine serum (FBS, Solarbio) and 1% penicillin–streptomycin (Solarbio) was utilized for the culture of the cells at 37°C in an incubator containing 5% CO_2_.

### 2.3 Cell transfection

H1299 cells and A549 cells at logarithmic growth stage were collected. After being diluted into 2 × l0^6^ cells/mL, the cells were seeded in a 6-well plate. The cells were transfected after cell fusion degree of 50–60%. GenePharma (Shanghai, China) designed and synthesized circ_0067741 small interfering RNA (si-circ_0067741), overexpressed plasmid of circ_0067741, LATS1 small interfering RNA (si-LATS1), miR-183-5p inhibitor, miR-183-5p mimics and their corresponding negative controls. Lipofectamine 2000 (Invitrogen, USA) transfection kit was applied to transfect the above plasmids into the cells. After 6-h culture, a complete medium replaced the original medium. And then the cells were collected 48 h after transfection.

### 2.4 qRT-PCR

After being extracted by Total RNA extraction kit (Tiangen, China), the total RNA from cells and tissues was placed at the temperature of – 80°C. Further, based on the instructions of the reverse transcription PCR kit (Takala, Japan), the reverse transcription was performed for the synthesis of cDNA, and the synthesized cDNA was tested for the concentration and purity. The quantitative PCR of cDNA was performed using SYBR Green qPCR kit (Takala, Japan). And 2^−ΔΔCt^ method was adopted for the data analysis [21]. The primer sequences used are displayed in Table 1.

**Table 1.** qRT-PCR primer sequences

Gene name	Primer sequences
circ_0067741	F 5’-CTACCAGCCAAACACCGCT-3’
	R 5’-TCCAGGAATCTGAAGGACCCA-3’
miR-183-5p	F 5’-GCGGCTATGGCACTGGTAGAA-3’
	R 5’-GTGCAGGGTCCGAGGTATTC-3’
LATS1	F 5’-AAACCAGGGAATGTGCAGCAA −3’
	R 5’-CATGCC TCTGAGGAACTA AGGA −3’
U6	F 5’-CGCTTCGGCAGCACATATAC-3’
	R 5’-TTCACGAATTTGCGTGTCATC-3’
GAPDH	F 5’- GGAGAT TACTGCCCTGGCTCCTA-3’
	R 5’-GACTCATCGTACTCCTGCTTGCTG −3’

### 2.5 MTT assay

After the collection of transfected H1299 cells and A549 cells, the cell concentration was adjusted to 1 × 10^4^ cells/ml. Then the cells were seeded in a 96-well microplate. After the cells were incubated for 24, 48 and 72 h, respectively, MTT solution for 20 μl of 5 mg/ml was placed in each well. Another 3-h incubation was then conducted at 37°C. Besides, 150 μl DMSO was put in each well after the supernatant was aspirated. Then the samples were mixed well at ambient temperature for 5 min, and a microplate reader was utilized for the test of the absorbance value at 490 nm.

### 2.6 Cell cloning experiments

The transfected cells were taken out and routinely digested to prepare for cell suspension. Then, a 6-well plate (2500 cells/well) was adopted to seed the cells. Further, 2 weeks after colony formation, 4% paraformaldehyde was utilized to fix the cells, and crystal violet was used for staining. Under an inverted microscope, the colonies (the numbers of cells 50 cells) were counted.

### 2.7 Apoptosis

H1299 and A549 apoptosis rate was checked by Annexin V-allophycocyanin (APC) apoptosis detection kit (BD Pharmingen, San Jose, USA). After collecting and washing with pre-chilled PBS, the suspension of the transfected cells was performed through 1 × Binding Buffer. Then 70% ethanol was utilized to fix the cells. Further, Annexin V and PI were added for the staining. Within 1 h, the apoptosis was determined through FACScan flow cytometry system (Becton Dickinson, San Diego, CA, USA).

### 2.8 Transwell cell migration and invasion assay

For the detection of cell migration, a serum-free medium was set up for the re-suspension of the cells. After re-suspension, the serum-free medium was placed in the upper chamber of the Transwell, and a complete medium was placed in the lower chamber. When cell invasion was detected, the matrigel was taken out from −20°C, and then was placed in the environment at 4°C overnight for melting. Then, according to the proportion of 1:6, the matrigel was diluted in the serum-free medium at 4°C. Subsequently, 100 μl of diluted matrigel was put in the upper chamber. And the upper chamber was then placed in the environment at 37°C for 3–5 h for the concretion of the matrigel. Then, 500 μl complete medium was placed in the lower chamber and 100 μl cells were placed in the upper chamber. After all the cells were cultured for 48 h, 4% paraformaldehyde was used for fixing, and crystal violet for staining. Then, under a microscope, the cells were counted.

### 2.9 Tube formation test

After plating, 1-h incubation for matrigel was conducted at 37°C. After adding 100 μL of transfected A549 and H1299 cells to a 96-well plate, a 24-h culture was performed at 37°C with 5% CO_2_. Then under an inverted microscope (40 ×), the cells were observed and photographed.

### 2.10 Dual-luciferase reporter assay

The constructed circ_0067741 wild-type (circ_0067741-WT)/mutant (circ_0067741-MUT) and LATSA1 wild-type (LATS1-WT)/mutant (LATS1-MUT) dual-luciferase reporter vectors were co-transfected with NC mimics/miR-183-5p mimics in 293 T cells, respectively, through Lipofectamine 2000 transfection reagent (Life Technologies, USA). After transfection and 48-h culture, the cells were lysed at ambient temperature for 20 min, then the centrifugation was conducted for the collection of supernatant. Luciferase activity was determined using a luminometer after addition of luciferase substrate. Renilla luciferase activity acted as an internal control, and then the calculation of relative firefly luciferase activity was conducted [22].

### 2.11 Western blot

RIPA lysis solution (Gibco, USA) was utilized to lyse the cells in each group for 30 min. Then, the cells were broken by sonication in the ice bath. Subsequently, after centrifugation at 12,000 rpm for 15 min at 4°C, the supernatant proteins of the cells were collected. The protein concentration was determined using the BCA assay kit (Saimefei, USA). After separation through SDS-PAGE, the transfer of proteins to PVDF membranes was carried out. After blocking the membranes for 1 h with 5% skimmed milk at ambient temperature for 1 h, then at 4°C, primary antibody was added for incubation overnight. Subsequently, the membranes were cleaned twice through TBST, and another 1-h incubation at ambient temperature was conducted after adding diluted enzyme-labeled secondary antibody. Luminescent solution was dropped to display the strip image in the exposure instrument. Protein level was analyzed using β-actin as an internal control.

### 2.12 Statistical analysis

Three independent experiments were performed for all data, and all experimental data analysis was verified through SPSS 22.0 software (SPSS Inc, Chicago, IL, USA). The comparison between the two groups was determined with *T*-test, and for comparison in multiple groups, one-way analysis of variance was applied. Correlation analysis was performed using Pearson analysis. The results were presented by mean ± standard deviation (SD). *P*< 0.05 was considered statistically significant. Data were displayed by Graphpad 9.0 software.

## 3. Results

### 3.1 Downregulation of circ_0067741 in cells and tissues of patients with lung adenocarcinoma

**Figure 1.**
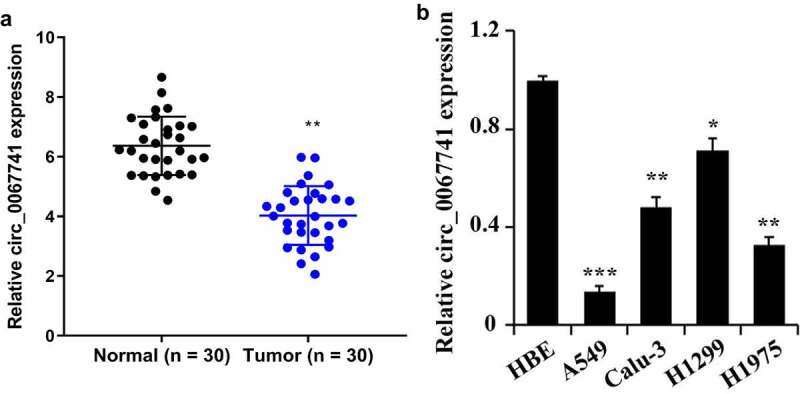
Expression of circ_0067741 in tissues and cells of patients with lung adenocarcinoma.

To study circ_0067741 expression in LUAD tissues and cells, qRT-PCR was utilized for the detection. The results displayed that the expression of circ_0067741 was decreased in the tissues of tumor group compared with the normal group (Figure 1a). In addition, circ_0067741 was also found to be significantly down-regulated in the LUAD cell lines compared with HBE cells. Among the above results, circ_0067741 expressions were highest in H1299 and lowest in A549 cells (Figure 1b). Therefore, subsequent experiments were carried out using H1299 and A549 cells as models. The results above suggested that circ_0067741 was involved in the growth of LUAD.

(a) Circ_0067741 expression in tissues of lung adenocarcinoma and its para-carcinoma tissues were checked through qRT-PCR, **P < 0.01; (b) qRT-PCR was adopted to check circ_0067741 expression in lung adenocarcinoma cell lines (Calu-3, H1299, H1975, A549) and human normal bronchial epithelial cells (HBE); *P < 0.05, **P < 0.01 and ***P < 0.001 *vs*. HBE group. All experiments were repeated three times.

### 3.2 Inhibition of circ_0067741 on the growth and development of lung adenocarcinoma cells

**Figure 2.**
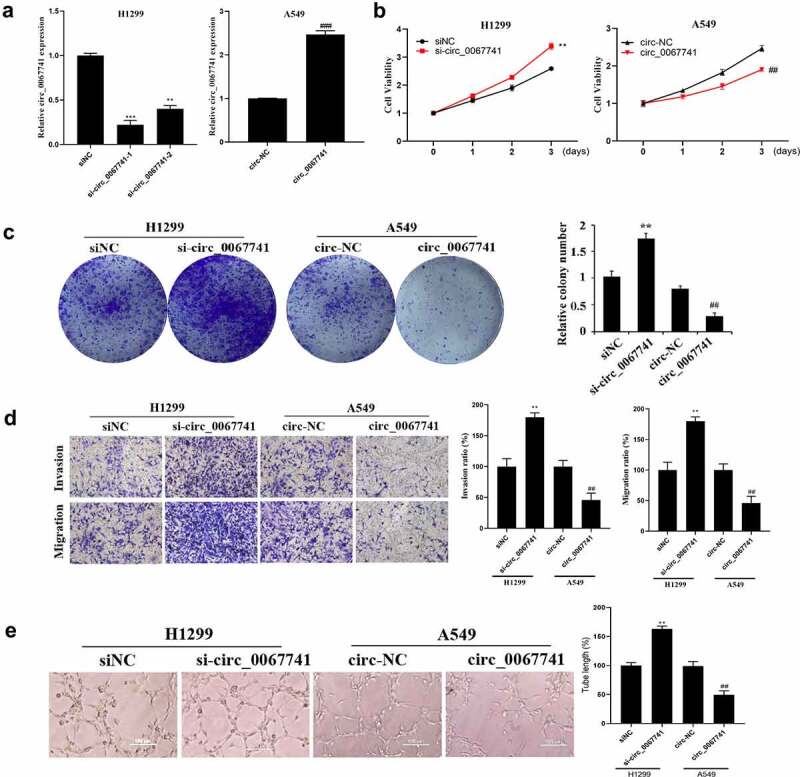
Effect of circ_0067741 on the growth and development of lung adenocarcinoma cells.

**Figure 3.**
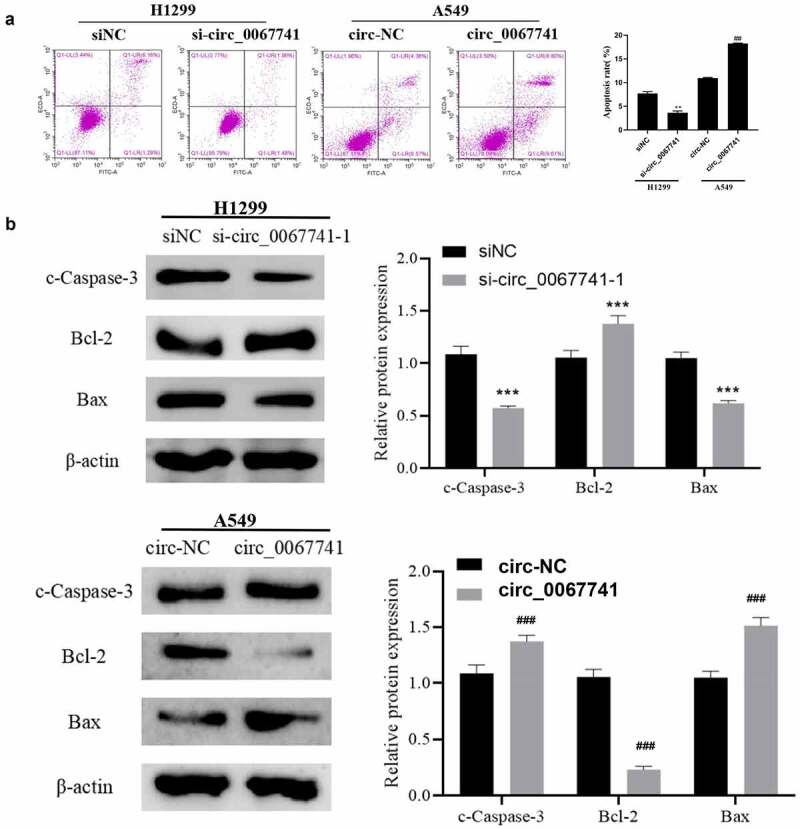
Effect of circ_0067741 on apoptosis of lung adenocarcinoma cells.

For the further understanding of the influence of circ_0067741 on LUAD cells, circ_0067741 was overexpressed or knocked down. The results showed that both si-circ_0067741-1 and si-circ_0067741-2 could reduce the expression of circ_0067741 in H1299 cells, and si-circ_0067741-1 was more effective. And overexpression of circ_0067741 significantly up-regulated circ_0067741 expressions in A549 cells (Figure 2a), which indicated successful transfection. In addition, after the knockdown of circ_0067741, the proliferation, invasion, viability, migration, and angiogenesis ability of H1299 cells were notably increased. However, after overexpression of circ_0067741, A549 cells had the opposite cellular processes such as proliferation, viability, invasion and migration (Figure 2b–e). Meanwhile, knockdown of circ_0067741 reduced the quantities of apoptotic cells (Figure 3a), and inhibited apoptosis-related proteins c-Caspase-3 expression and Bax and up-regulated Bcl-2 expression (Figure 3b). In summary, circ_0067741 could affect the development of LUAD cells.

After transfection, (a) qRT-PCR for the verification of circ_0067741 expression in each group of A549 and H1299 cells. (b) The proliferation ability of H1299 and A549 cells was measured by MTT. (c) Cell cloning assay was utilized to check the cell viability of A549 and H1299 cells. (d) Transwell was adopted for the detection of the invasion and migration ability of A549 and H1299 cells. (e), Tube formation assay was conducted for the test of the angiogenesis of each group of H1299 and A549 cells; Error bars stand for the mean ± SD of three independent measurements. **P < 0.01 *vs*. siNC group, ##*P* < 0.01 *vs*. circ-NC group.

After transfection, (a) The apoptosis of H1299 and A549 cells was determined with flow cytometry. (b) Western blot was utilized to check apoptotic phase protein expression of A549 and H1299 cells; Error bars stand for the mean ± SD of three independent measurements. **P < 0.01 and ***P < 0.001 *vs*. siNC group, ##*P* < 0.01 and ###*P* < 0.001 *vs*. circ-NC group.

### 3.3 Circ_0067741 can sponge miR-183-5p in lung adenocarcinoma cells

**Figure 4.**
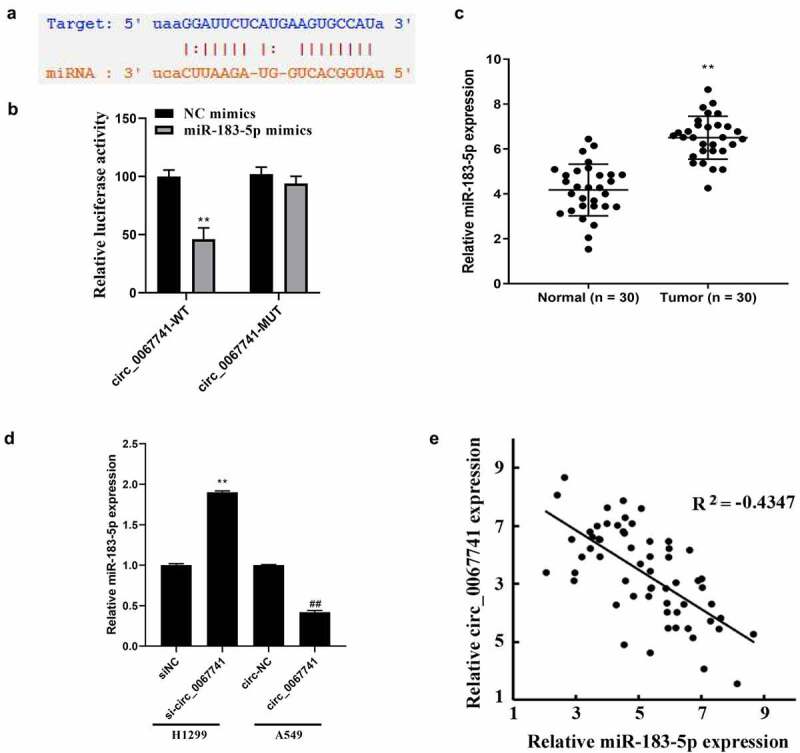
Circ_0067741 targets and regulates miR-183-5p expression in lung adenocarcinoma.

ENCORI database (https://starbase.sysu.edu.cn/) was carried out for prediction of the target genes of circ_0067741, and the results disclosed that circ_0067741 interacted with miR-183-5p (Figure 4a). Dual-luciferase reporter assay result also indicated a notable reduction in the luciferase activity of circ_0067741-WT due to overexpression of miR-183-5p, while miR-183-5p overexpression had no impact on the luciferase activity of circ_0067741-MUT (Figure 4b). Also, miR-183-5p expression was raised in LUAD tissues (Figure 4c). Besides, miR-183-5p expression was increased or decreased after silencing and overexpression of circ_0067741 in H1299 and A549 cells, respectively (Figure 4d). Correlation analysis also displayed that circ_0067741 was negatively associated with the miR-183-5p in LUAD (Figure 4e). The above results stated that circ_0067741 targeted and regulated miR-183-5p expression in LUAD.

(a) ENCORI database predicted the targeting relationship between miR-183-5p and circ_0067741. (b) The combination of circ_0067741 and miR-183-5p was checked through dual-luciferase reporter assay; **P < 0.01 *vs*. NC mimics group. (c) qRT-PCR was for miR-183-5p expression detection in lung adenocarcinoma tissues; **P < 0.01. (d) qRT-PCR was utilized to test miR-183-5p expression in A549 and H1299 cells after transfecting; **P < 0.01 *vs*. siNC group, ##*P* < 0.01 *vs*. circ-NC group. (e) The relationship between circ_0067741 and miR-183-5p was analyzed through correlation analysis. Error bars stand for the mean ± SD of three independent measurements.

### 3.4 Circ_0067741 can regulate LATS1 to activate Hippo/YAP pathway through miR-183-5p

**Figure 5.**
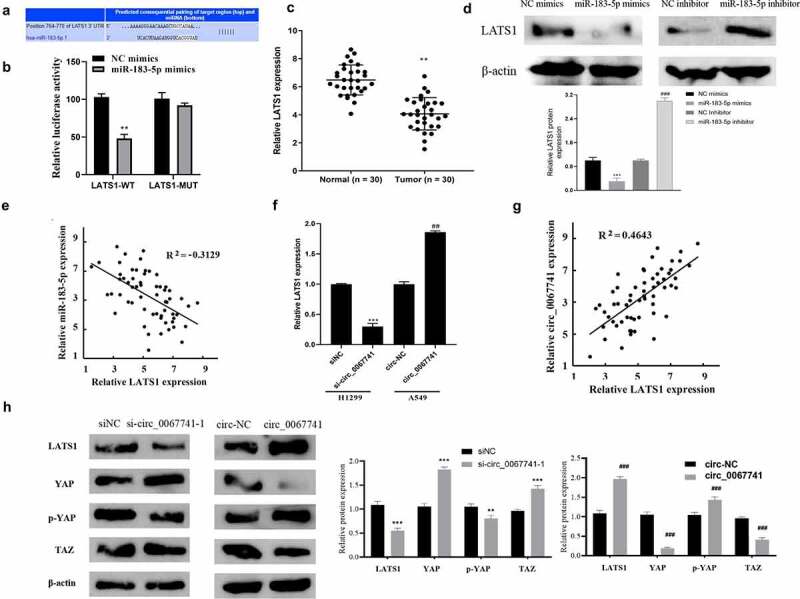
Effect of circ_0067741 on LATS1 expression and Hippo/YAP pathway.

TargetScan database (https://targetscan.org/vert_72/) was applied for the prediction of miR-183-5p potential target genes, and the results showed an interactive relationship between LATS1 and miR-183-5p (Figure 5a). According to dual-luciferase gene reporter assay results, the luciferase activity of the cells co-transfected with miR-183-5p mimics and LATs1-WT was significantly decreased, but the luciferase activity of the transfected LATS1-MUT cells was not impacted, confirming the targeting relationship between LATS1 and miR-183-5p (Figure 5b). LATS1 was further found to be declined in LUAD tissues (Figure 5c). Besides, after miR-183-5p was over-expressed, LATS1 protein expression in cells was significantly reduced, while knockdown of miR-183-5p reversed above results (Figure 5d). Correlation analysis also showed a negative association between LATS1 and miR-183-5p expression in LUAD (Figure 5e). And LATS1 expression was obviously declined after circ_0067741 was knocked out in LUAD, while the overexpression of circ_0067741 gave the opposite result (Figure 5f). Correlation analysis suggested that circ_0067741 was associated with LATS1 positively (Figure 5g).

(a) TargetScan database was used for the prediction of the targeting relationship between LATS1 and miR-183-5p. (b) dual-luciferase reporter assay for the correlation between miR-183-5p and LATS1; **P < 0.01 *vs*. NC mimics group. (c) qRT-PCR for checking LATS1 expression in lung adenocarcinoma tissues; **P < 0.01 *vs*. Normal group. (d) Western blot to measure LATS1 expression in the cells after overexpression or knockdown of miR-183-5p; ***P < 0.001 *vs*. NC mimics group, ###*P* < 0.001 *vs*. NC-inhibitor group. (e) Correlation analysis for the relationship between miR-183-5p and LATS1. (f), qRT-PCR for testing LATS1 expression in the cells after knockdown or overexpression of circ_0067741; ***P < 0.001 *vs*. siNC group, ##*P* < 0.01 *vs*. circ-NC group. (g) Correlation analysis of the relationship between circ_0067741 and LATS1. (h) Western blot was carried out to determine Hippo/YAP pathway-related protein expression; **P < 0.01 and ***P < 0.001 *vs*. siNC group, ###*P* < 0.001 *vs*. circ-NC Group. Error bars stand for the mean ± SD of three independent measurements.

Further knockdown or overexpression of circ_0067741 was performed to detect Hippo/YAP pathway-related proteins closely associated with LATS1 proteins. The results displayed that after the knockdown of circ_0067741, LATS1 and p-YAP expression was inhibited, YAP and TAZ expression was promoted, and Hippo/YAP pathway activation was suppressed; while the results were reversed when circ_0067741 was overexpressed (Figure 5h). The results above revealed that circ_0067741 could target and regulate LATS1 protein to activate Hippo/YAP pathway through miR-183-5p.

#### 3.5 Downregulation of LATS1 Reverses the Effect of circ_0067741 on the Progression of Lung Adenocarcinoma Cells

**Figure 6.**
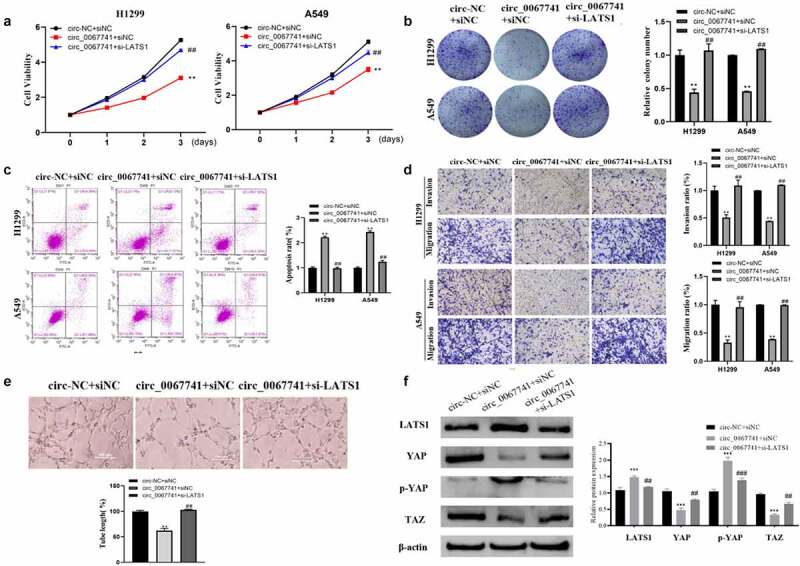
Effect of LATS1 downregulation on the progression of lung adenocarcinoma cells affected by circ_0067741.

To further determine whether circ_0067741 could regulate the Hippo/YAP pathway to affect the process of LUAD through the miR-183-5p/LATS1 axis, a cell reversion assay was performed. The results presented a notable decrease in the proliferation, viability, invasion, migration, and tube formation ability of LUAD cells, and an increase in the apoptosis rate when circ_0067741 was overexpressed; on the above basis, the proliferation, viability, invasion and migration ability, and tube formation ability of the cells were significantly increased, and the apoptosis rate of cells was notably reduced after downregulation of LATS1 (Figure 6a–e). In addition, the overexpression of circ_0067741 also promoted p-YAP expression and LATS1, and suppressed YAP and TAZ level. The above results could be reversed after further downregulation of LATS1 (Figure 6f). All in all, circ_0067741 could be a sponge of miR-183-5p to increase LATS1 expression and affect the development of LUAD.

(a) MTT for detecting H1299 and A549 proliferation. (b) Cell cloning assay was adopted for the detection of H1299 and A549 viability. (c) Transwell assay for the invasion and migration ability of H1299 and A549. (d) Tube formation assay for determining the angiogenesis of H1299 and A549. (e) Flow cytometry to check the apoptosis of H1299 and A549 cells. (f) Western blot was applied to check Hippo/YAP pathway-related protein expression in lung adenocarcinoma cells. All experiments were repeated three times. **P < 0.01 and ***P < 0.001 *vs*. circ-NC + siNC group, ##*P* < 0.01 *vs*. circ_0067741 + siNC group.

## 4. Discussion

Lung cancer is one of the major causes for the depressed survival rate in cancer patients. Compared with other tumors, the incidence and mortality of lung cancer are higher [3]. At the same time, the treatment of lung cancer still suffered great challenges on account of the metastasis and poor prognosis [23,24]. It is revealed that aberrant expression of transcriptional regulatory genes is closely associated with the emergence and malignant behavior of numerous cancers [25,26]. CircRNA can regulate gene expression by targeting sponge-adsorbed miRNAs [10,27] and then to be involved in the tumorigenesis and development. It has been reported that circ_0020850 can regulate the miR-195-5p/IRS2 axis to inhibit LUAD development [14]. The above results suggest that circRNA has potential value as a diagnostic marker for LUAD. Circ_0067741 is considered as a newly discovered circRNA recently. In this study, circ_0067741 expression was declined significantly in LUAD cell lines and tissues, and circ_0067741 inhibited LUAD cell invasion, migration, proliferation and angiogenesis, and promoted apoptosis. It was further found that circ_0067741 could target miR-183-5p expression; miR-183-5p expression was raised notably in LUAD cell lines and tissues, which was associated with circ_0067741 expression negatively. It is reported miR-183-5p has joined in the proceeding of various cancers, for example, miR-183-5p can play a cancer suppression role in lung cancer by regulating PIK3CA [28]. However, some studies have also found that miR-183-5p suppresses the phosphorylation of p53 to activate the AKT signaling pathway, and then to promote tumor metastasis and development in non-small cell lung cancer [29]. It can be seen that miR-183-5p is able to control different target genes in tumors, with great differences in biological effects. The above studies were consistent with this study.

LATS1, also known as the large tumor suppressor gene, is located at the position 24–25 of the long arm of chromosome 6, which belongs to the dbf2 nuclear protein kinase family. LATS1 has a very high similarity in structure and function with LATS2, which is one of the core sections of the Hippo signaling pathway [30]. It has been shown that there is a close relationship between abnormally expressed LATS1 and the abnormal proliferation and apoptosis of various cancer cells such as gastric cancer, skin cancer and metastatic prostate cancer [31,32]. Some miRNAs regulate the Hippo signaling pathway to participate in tumor development. For instance, the miR-130a-YAP positive feedback loop promotes tumor cell proliferation [33], while miR-129 inhibits ovarian cancer development by inhibiting the Hippo signaling effectors TAZ and YAP [34]. In this study, it was also found that there was a targeting relationship between LATS1 and miR-183-5p, while miR-183-5p could activate the Hippo/YAP pathway by regulating LATS1 expression. And the impact of circ_0067741 on the progression of LUAD cells and the role in Hippo/YAP pathway could be reversed after downregulation of LATS1 expression. On the basis of the above results, it was estimated that circ_0067741 could regulate the biological behavior of LUAD cells, involving LUAD cells proliferation, viability, invasion, migration, angiogenesis ability and apoptosis. Specifically, circ_0067741 sponged miR-183-5p to regulate LATS1 expression to activate the Hippo/YAP pathway, thereby regulating the biological behaviors of LUAD cells. This paper offered a new clue to the research of the mechanism of LUAD cells development.

## 5 Conclusion

In summary, this study has showed that circ_0067741 regulates the biological behavior of LUAD cells by sponging miR-183-5p, up-regulating LATS1 expression, and activating the Hippo/YAP pathway. This research has provided a reference for the role and mechanism of circ_0067741 in LUAD, revealing the potential of circ_0067741 as the target to diagnose and treat LUAD early.

## Data Availability

The data used to support the findings of this study are included within the article. Further inquiries can be directed to the corresponding author.
